# Epidemiology of Cholera in the Philippines

**DOI:** 10.1371/journal.pntd.0003440

**Published:** 2015-01-08

**Authors:** Anna Lena Lopez, Lino Y. Macasaet, Michelle Ylade, Enrique A. Tayag, Mohammad Ali

**Affiliations:** 1 University of the Philippines Manila-National Institutes of Health, Philippines; 2 Department of Health, Manila, Philippines; 3 Department of International Health, Johns Hopkins Bloomberg School of Public Health, Baltimore, Maryland, United States of America; Massachusetts General Hospital, United States of America

## Abstract

**Background:**

Despite being a cholera-endemic country, data on cholera in the Philippines remain sparse. Knowing the areas where cholera is known to occur and the factors that lead to its occurrence will assist in planning preventive measures and disaster mitigation.

**Methods:**

Using sentinel surveillance data, PubMed and ProMED searches covering information from 2008–2013 and event-based surveillance reports from 2010–2013, we assessed the epidemiology of cholera in the Philippines. Using spatial log regression, we assessed the role of water, sanitation and population density on the incidence of cholera.

**Results and Discussion:**

We identified 12 articles from ProMED and none from PubMed that reported on cholera in the Philippines from 2008 to 2013. Data from ProMed and surveillance revealed 42,071 suspected and confirmed cholera cases reported from 2008 to 2013, among which only 5,006 were confirmed. 38 (47%) of 81 provinces and metropolitan regions reported at least one confirmed case of cholera and 32 (40%) reported at least one suspected case. The overall case fatality ratio in sentinel sites was 0.62%, but was 2% in outbreaks. All age groups were affected. Using both confirmed and suspected cholera cases, the average annual incidence in 2010–2013 was 9.1 per 100,000 population. Poor access to improved sanitation was consistently associated with higher cholera incidence. Paradoxically, access to improved water sources was associated with higher cholera incidence using both suspected and confirmed cholera data sources. This finding may have been due to the breakdown in the infrastructure and non-chlorination of water supplies, emphasizing the need to maintain public water systems.

**Conclusion:**

Our findings confirm that cholera affects a large proportion of the provinces in the country. Identifying areas most at risk for cholera will support the development and implementation of policies to minimize the morbidity and mortality due to this disease.

## Author Summary

Cholera has been increasingly reported in the past decade. It is most feared because of its tendency to spread rapidly resulting in deaths in a short time, if appropriate treatment is not provided. For fear of trade and travel sanctions, countries were disinclined to report cholera, unless large outbreaks ensued. Although countries in Asia have been reporting cholera, it is believed that more cases are not being identified and instead being reported as acute watery diarrhea. Cholera is endemic in the Philippines however data on cholera in the country remained sparse, until 2008 when surveillance was strengthened. From 2008 to 2013, 42,071 suspected and confirmed cholera cases were reported in 87% of provinces and metropolitan areas in the country, confirming the endemicity of cholera in the Philippines. Poor access to improved sanitation was associated with cholera. On the other hand despite access to improved water sources, cholera remains to be seen. The latter is most probably due to the breakdown and non-chlorination of water systems. We identified areas where cholera has been known to occur in the Philippines, this will assist in the development and implementation of policies to minimize the morbidity and mortality due to this disease.

## Introduction

Cholera strikes fear in many areas as it rapidly spreads and results in death within hours if appropriate therapy is not provided. The disease particularly affects communities with poor water and sanitation infrastructure leading to outbreaks, which if not managed properly, have deadly consequences. In areas with already limited health facilities, cholera outbreaks can easily overwhelm health care systems further compromising provision of appropriate health care to patients with the disease.

Clean water and sanitation have largely purged cholera from the developed world, yet cholera continues to cause substantial suffering in a large portion of the globe. But as more people move to the cities with teeming slums and population displacement due to wars, famine or natural disasters occur, cholera will continue to be a problem as long as access to clean water and sanitation is not assured.

After an absence of more than 25 years, cholera was again reported in the Philippines in 1961. Following the first reports of cases of *Vibrio cholerae* O1 biotype El Tor in Manila in 1961, the Philippines has been considered as a cholera endemic country [1]. It is estimated that there are ∼18 million individuals at risk for cholera in the Philippines with an estimated annual incidence of 0.1/1,000 persons at risk [2]. However, data on cholera in the Philippines remain sparse, since no systematic diarrheal disease surveillance existed that required laboratory confirmation prior to 2008.

To comply with the 2005 International Health Regulations (IHR), the National Epidemiology Center (NEC), under the Philippines' Department of Health (DOH) implemented the Philippine Integrated Disease Surveillance and Response (PIDSR) system, establishing a network of disease surveillance units that monitors 23 diseases or conditions, including outbreak prone diseases such as cholera. The PIDSR was launched in 2008 and as part of PIDSR, suspected cholera cases in surveillance units were reported and investigated, including laboratory confirmation of etiologic agents [3]. At the same time, suspected outbreaks were reported and investigated in the Event-based Surveillance and Response (ESR), established in 2010 also to comply with the 2005 IHR.

With these new sources of information, we aimed to assess the epidemiology and the geographic distribution of cholera in the country as well as identify factors that may be associated with the occurrence of the disease. This information will be used to support future policies and program implementation, including the possible use of oral cholera vaccines (OCV) in targeted areas, with the goal of mitigating the impact of cholera in the country.

## Materials and Methods

### The country

The Philippines is an archipelago of ∼7,107 islands and is located in the western Pacific Ocean in Southeastern Asia. It is bordered by bodies of water: the Bashi Channel to the north, the Pacific Ocean to the east, the Sulu and Celebes Seas to the south and the South China Sea to the west. As of the 2010 census, the country had a population of 92,337,852, with ∼12% of its population residing outside of the Philippines. The country has a young population and 44% are younger than 14 years of age. It has two seasons, the rainy season, from June to November and the dry season, from December to May [4].

### Definitions

The PIDSR and ESR uses the definitions for cholera [5] based on the WHO definition for cholera [6]. A suspected cholera case is defined as: (a) in an area where the disease is not known to be present, a patient aged 5 years or more with severe dehydration or dies from acute watery diarrhea; or (b) in an area where cholera is endemic, a patient aged 5 years or more with acute watery diarrhea with or without vomiting; or (c) in an area where there is a cholera epidemic, a patient with acute watery diarrhea, with or without vomiting. A confirmed cholera case is a suspected case wherein *Vibrio cholerae* O1 or O139 is identified in the stool.

The WHO recommends the laboratory confirmation of the first cases to ascertain that there is a cholera outbreak. Once the outbreak is confirmed, the clinical case definition is used to detect additional cholera cases and provide treatment [6]. Cases were assessed as having suspected or confirmed cholera based on the NEC's classification. Only those with *V. cholerae* O1 or O139 in the stools were considered as laboratory-confirmed cases. We also restricted the number of suspected cases by excluding those whose stool cultures identified other organisms, whether pathogenic or not.

### Sources of data

Data on cholera in the Philippines were obtained from two main sources of information: the PIDSR and the ESR systems (For details, see S1 Table), both managed by the NEC. For cholera, a line-list, case-based, passive surveillance is conducted at the sentinel hospital wherein a minimum set of data is collected. Anonymized data from the cholera line-list, case-based surveillance from 2008 to 2013 was obtained from NEC.

The ESR system of the NEC, which became operational from 2010, detects events that potentially impact public health. Initial and follow-up ESR reports were obtained and reviewed. Diarrheal and acute gastroenteritis reports from 2010 to 2013 were identified and were included as confirmed if follow-up reports indicate that the events were due to *V. cholerae O1 or O139*. Typical of outbreak reports, patient characteristics such as age and gender, the presenting symptoms and signs, locations of the patient's residence and reporting facility and dates of first presentation of cases were recorded at the municipality level (if available) or up to the provincial level in a separate spreadsheet. ESR reports were counterchecked with the PIDSR database to avoid duplication of cases.

The Field Epidemiology Training Program (FETP) of NEC conducted further investigations in some outbreaks to assess the causality of outbreaks. Reports on factors associated with the occurrence of the outbreaks from these investigations were tabulated.

NEC reports were supplemented by literature review using the search words “cholera” and “Philippines” in PubMed. Furthermore, data from an alternative database, Program for Monitoring Emerging Diseases (ProMED), an online forum for infectious disease specialists, microbiologists and public health officials established in 1994 and administered by the International Society for Infectious Diseases since 1999 [7] were also reviewed to ensure that no reports were missed. To avoid double counting of cholera cases and deaths, reports were crosschecked with events in the ESR and potential cases in PIDSR based on the dates and the locations (communities and provinces) where the patients came from. Potential overlapping reports were reviewed and the ones with more complete information were included. Information from local publications was identified through Google search and review of local journals.

Isolates were submitted to the national reference laboratory at the Research Institute for Tropical Medicine (RITM). Data from the Antimicrobial Resistance Surveillance Program (ARSP) from 2008 to 2011 as well as published reports from the ARSP were identified.

To assess the role of water, sanitation and population density in cholera in the Philippines, data on access to clean water and sanitation were obtained from 2011 [8]. To obtain cholera incidence, population by city or municipality was obtained from the 2010 Philippines census [4].

### Mapping

All cases obtained from PIDSR (2008–2013), ESR (2010–2013) and ProMED (2008–2013) were collected and tabulated according to the municipalities and cities where the patients came from in a Microsoft Excel (Seattle USA) spreadsheet. Cases were then entered into an ArcGIS software (ESRI, California, USA) to map the sites wherein cholera has been reported. Average annual incidence of both suspected and confirmed cholera cases were obtained per 100,000 population and mapped.

### Statistical analysis

To evaluate the role of water, sanitation and population density and cholera, we used a spatial lag regression (SLR) model implemented in GeoDA^™^ version 1.4.6, assuming that spatial dependencies existed among the levels of the dependent variable, i.e., cholera transmission in one location was affected by the same at the nearby locations. The SLR model is a maximum likelihood estimate that uses a spatially lagged dependent variable. Formally, this model is y =  ρWy + xβ +ε, where y is a vector of observations of the dependent variable (annual cholera incidence rate), Wy is a spatially lagged dependent variable for weight matrix W, x is a matrix of observations of the explanatory variables, ε is the vector of the independently and identically distributed error terms, and ρ and β are parameters [9]. The spatial weights were constructed based on three orders of Queen Contiguity (contiguity based on shared border or vertices). Queen contiguity was selected over Rook Contiguity (contiguity based on shared border only) because the absolute length of two municipalities' shared border was less important than the proximity of the municipalities.

### Ethics

The University of the Philippines Manila Research Ethics Board approval was obtained before the study was initiated. Because this study conducted analysis of existing government data with all identifying information deleted from the database prior to analysis, an exemption from review was granted.

## Results

Annually, from 2008 to 2013, 78 to 96 sentinel hospitals and DRUs reported a total of 29,163 cases to PIDSR. After excluding updates from the same outbreaks, there were 106 ESR reports of suspected cholera outbreaks from 2010 to 2013. A search in ProMED using the search terms “cholera” and “Philippines” identified 12 articles that reported on 6,829 cases from 2008 to 2013. Among these only 3,171 cases were not overlapping with the ESR and the PIDSR reports. Using the same search terms in PubMed revealed 85 articles, none reported on cholera cases from 2008 to 2013. The WHO reports that are published annually relied on data from DOH and thus were excluded to prevent double counting. Two locally published reports on the ARSP were identified and included.

**Figure 1:**
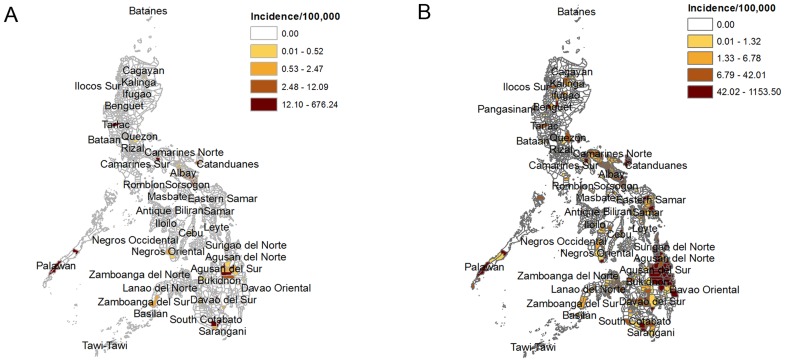
Average annual cholera incidence (per 100,000 population), by municipality of confirmed (1A) and both suspected and confirmed cholera cases (1B) from all sources.

There were 42,071 suspected and confirmed cholera cases from 2008 to 2013 from PIDSR, ESR and ProMED, among which 5,006 (12%) were confirmed cholera cases. Table 1 shows the annual distribution of suspected and confirmed cholera cases. Since ESR only started in 2010, data from 2010 to 2013 was used to calculate the average annual incidence. Based on suspected and confirmed cholera cases the incidence was 9.1 per 100,000 population. However, when analyzed by municipality or city, the incidence ranged from 0 to 2,678.1 per 100,000. The highest incidence was reported in the 2012 outbreak in Virac, Catanduanes affecting 1,793 people, the highest number reported from one city alone. 38 (47%) of 81 provinces and metropolitan regions reported at least one confirmed case of cholera, 32 (40%) reported at least one suspected case while 11 (14%) reported no case at all. Fig. 1a and 1b show the average annual incidence, by municipality, of confirmed and both suspected and confirmed cholera cases from all sources.

**Table 1:** Suspected and confirmed cholera cases, estimated annual incidence from 2008 to 2013.

Year	ESR N (% confirmed)*	PIDSR	ProMED	Total Cases	Incidence (per 100,000 population)
2008	NA†	1463	1581	2981	3.23
2009	NA†	5521	124	5645	6.11
2010	1467 (16.2%)	4248	30	5745	6.22
2011	2792 (50.8%)	6977	-	9769	10.58
2012	4487 (51.9%)	7029	1331	12847	13.91
2013	991 (38.1%)	3925	168	5084	5.50
Average incidence (2010–2013)‡					9.1

*Per cent culture confirmed among cases.

† NA – Not available.

‡ Average incidence calculation includes data from 2010 to 2013 since ESR began in 2010.

Among the 29,163 suspected and confirmed cholera cases in PIDSR from 2008 to 2013, 4,088 cases underwent laboratory testing. Out of these cases that underwent testing, 602 (15%) were laboratory confirmed or 2% of all cases in PIDSR. Information on individual cases allowed identification of age and sex distribution of confirmed and both suspected and confirmed cases reported in the PIDSR (Table 2). Majority of cases did not undergo laboratory testing (71% in 2008, 88% in 2009, 86% in 2010, 84% in 2011 and 2013 and 83% in 2012). There were 187 deaths from 2008–2013, with annual case fatality ratio (CFR) ranging from 0.21% in 2008 to 0.72% in 2011 and the overall CFR was 0.62%. The number of deaths and case fatality ratio by age group and sex is in Table 2. The highest case fatality ratio was seen among children under 5 years of age.

**Table 2:** Age and sex distribution of suspected and confirmed cholera cases from PIDSR, 2008–2012.

Characteristics	Suspected and confirmed cases* *n (%)*	Confirmed cases† *n (%)*	No. of deaths among suspected and confirmed cases‡ *n (%)*	Case Fatality Ratio§
***Age group***				
** 0-<5**	4602 (16%)	193 (32%)	47 (26%)	1.02%
** 5-<15**	5615 (16%)	175 (29%)	35 (19%)	0.62%
** 15+**	18927 (65%)	230 (38%)	100 (55%)	0.53%
***Sex***				
** Male**	14876 (49%)	329 (55%)	103 (49%)	0.69%
** Female**	15406 (51%)	273 (45%)	86 (54%)	0.56%

*Out of 29,144 cases with data on age and 30,282 with data on sex.

† Out of 598 cases with data on age and 602 with data on sex.

‡ Out of 182 cases with data on age and 189 with data on sex.

§ Out of 182 cases with data on age and 187 cases with data on sex.

**Figure 2:**
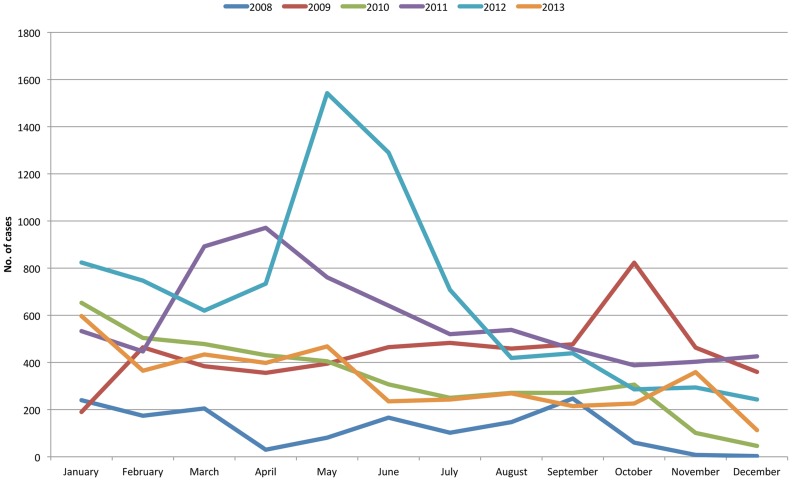
Seasonality of suspected and confirmed cholera cases in PIDSR, by month and year.

Seasonality of cholera was obtained by tabulating cases from the PIDSR dataset by month and year (see Fig. 2). Suspected and confirmed cases were reported every month, and while no distinct seasonality was seen every year, increases in the number of cases were noted between the months of March to June although less consistently, smaller peaks were seen in September to November.

From 2010 to 2013, out of 106 suspected outbreaks reported in ESR, microbiologic confirmation was performed in 64 (60%), among which 29 (45%) were confirmed *V. cholerae* O1 outbreaks affecting 5,402 individuals (Table 3). There were 77 suspected cholera outbreaks affecting 4,335 individuals in the Philippines. The largest confirmed cholera outbreak, affecting the whole province of Catanduanes and other provinces in the Bicol region was recorded in June 2012. A state of calamity was declared in Catanduanes, following a large cholera outbreak involving the capital, Virac and several towns with 3,390 possible cases and 19 deaths [10]. This resurgence of cholera was associated with problems in sanitation, water and poor hygiene [11]. Prior to this outbreak, all municipalities in the province of Catanduanes had reported suspected cholera cases in PIDSR since its implementation in 2009 in the province, however none had been laboratory-confirmed. A diarrheal death was identified in a 67 year old in 2009 in PIDSR and none thereafter. Virac, the capital of the province where the 2012 outbreak began had the most number of cases from 2009–2011. The second biggest outbreak was in 2011, when several communities with indigenous people were affected in a remote area in Palawan, affecting 1,226 individuals.

**Table 3:** Cholera confirmed outbreaks reported, cases and deaths identified and ages affected, from ESR 2010–2013.

Year	No. of confirmed cholera outbreaks	No. of suspected cases per outbreak, Range (Median, Mean)	No. of deaths	Case Fatality Ratio, Range (Median, Mean)	Age range
2010	5	14–166 (19, 48)	5	0 to 14.3% (4.5%, 6.1%)	1 yr to 74 yrs.
2011	10	11–563 (58, 142)	65	0 to 23.6% (3.4%, 5%)	1 mo to 90 yrs
2012	8	1–1793 (55, 353)	30	0 to 4.3% (0.8%, 1.4%)	1 mo to 63 yrs
2013	6	22–135 (68, 69)	5	0 to 10.8% (0.4%, 2%)	4 mos to 83 yrs

There were 112 reported deaths in the 29 cholera confirmed outbreaks reported. The highest number of deaths occurred in Palawan, with 29 deaths each on two separate outbreaks. CFR ranged from 0 to 23.6% (overall CFR- 2%), with the highest occurring in one outbreak in Rizal, Palawan. In all outbreaks, all age groups were affected.

There were seven FETP outbreak reports from 2008 to 2013 that were included (S2 Table). In all investigations, rectal swabs and water samples were obtained from a small subset of patients. In all seven outbreaks, *V. cholerae* O1 El Tor Ogawa was identified in rectal swabs of patients. Five out of the 7 investigated outbreaks occurred in areas where there were breakdowns in the water infrastructure. Contamination of water sources was identified in all.

From 2008 to 2011, there were 217 isolates of *V. cholerae* O1 in the ARSP. Among the 115 isolates further tested for serotyping, 110 were Ogawa and 5 were Inaba serotypes. In 2009, among 88 isolates, no organism was identified as resistant to tetracycline, cotrimoxazole or chloramphenicol [12]. In 2012, there were 35 *V. cholerae* isolates. Resistance ratios in 2011–2012 were 3.7% and 0.9% to tetracycline and chloramphenicol, respectively. No co-trimoxazole resistance was identified in 2011 and 2012 [13].

To assess the role of water, sanitation and population density to the incidence of cholera in the municipality or city, we initially fit the data in an ordinary least squares (OLS) regression model with weight matrix. The Jarque-Bera statistic yielded non-normality as well as high spatial correlation of the combined data sets for confirmed (Moran's I: 0.04, p = .004) and both confirmed and suspected cases (Moran's I: 0.02, p = .001). Based on these results we concluded that a spatial lag regression (SLR) model would be suitable for analyzing these datasets. The SLR model explained better total variation (R^2^) over the OLS model. However, the SLR has a pseudo-R^2^ that cannot be directly compared with the R^2^ of the OLS model. The proper measures of fit are the log-likelihood, Akaike Information Criterion (AIC), and the Schwarz Criterion (SC). The SLR model had increased log-likelihood compared to the OLS model. The AIC and the SC were lower in SLR model in comparison to the OLS model. These lower values for the SLR compared with the OLS models suggest an improved fit for the spatial lag specification.

Using confirmed cholera data only, the results of the SLR model with three orders of neighbor spatial weight showed significantly negative association (coefficient  = −0.0467, p = .049) (Table 4) between cholera incidence and the population's access to improved sanitation. In analysis of both confirmed and suspected cholera, a significantly negative association between cholera incidence and population's access to improved sanitation system (coefficient  = −.2292, p = .0004) was obtained. In addition, a significantly positive association between cholera incidence and population's access to improved water sources (coefficient  = .2563, p = .0004) was identified in the same analysis (Table 5). The spatial patterns of residuals were also analyzed by creating a Moran's I scatter plot. The test statistic for the spatial lag residuals of confirmed and confirmed plus suspected yielded Moran's I as 0.01 (p = .03) and.0004 (p = .39), respectively.

**Table 4:** Risk for incidence of cholera among municipalities in Philippines, 2010–2013, *using confirmed cases only*.

Variable	Coefficient	Std. Error	z-value	P-value
Spatially lagged annual cholera incidence rate for weight matrix	0.21	0.051	4.184	0.00002
Constant	2.55	1.854	1.376	0.17
Per cent of people had access to improved water sources	0.014	0.026	0.531	0.59
Per cent of people had access to improved sanitation system	−0.047	0.024	−1.97	0.049
Population density/Km^2^	−.000003	0.00017	−0.018	0.98

**Table 5:** Risk for incidence of cholera among municipalities in the Philippines, 2010–2013, *using confirmed and suspected cases*.

Variable	Coefficient	Std. Error	z-value	P-value
Spatially lagged annual cholera incidence rate for weight matrix	0.550281	0.038119	14.43558	<0.0001
Constant	−0.284633	5.003261	−0.056889	0.95
Per cent of people had access to improved water sources	0.256308	0.072556	3.532554	0.0004
Per cent of people had access to improved sanitation system	−0.229242	0.064808	−3.537204	0.0004
Population density/Km^2^	−0.000305	0.000459	−0.665382	0.50

## Discussion

We present the most extensive information on suspected and confirmed cholera cases in the Philippines in the past six years. Based on the analysis of data from 2010–2013, the annual incidence of suspected and confirmed cholera cases in the country was 9.1 per 100,000 individuals. This incidence is most likely lower than the true incidence since it is based on information from sentinel sites that did not consistently report and outbreak reports. Of the more than 500 sentinel sites, less than 20% reported cases. Furthermore, outbreaks were likely not all captured by the ESR since events that occur in far-flung areas rarely reach the DOH central office unless deaths occurred.

The incidence of suspected and confirmed cholera that we obtained is substantially lower than the incidence of reported acute watery diarrhea cases reported annually in the Field Health Service Information System (FHSIS). A major component of the DOH to enable it to better manage its nationwide health programs, the FHSIS obtains basic health seeking information and service delivery up to the level of barangays (villages) [14]. From 2008 to 2011, acute watery diarrhea remained as one of the top 10 leading causes of morbidity in the FHSIS [15]–[18]. In 2011 alone there were 210,700 cases reported (or 220 per 100,000 population) [18]. Since data from the FHSIS does not include laboratory results, the proportion of cholera among these acute watery diarrhea cases is unclear. Similar to other countries in Asia, the Philippines does not report acute watery diarrhea cases as cholera cases [19], [20].

Microbiologic analysis revealed that among those tested 96% belonged to the Ogawa serotype, similar to findings in other countries [21]–[23]. Data from the ARSP confirms the susceptibility of *V. cholerae* isolates to currently used first line antibiotics and do not require use of more expensive second line drugs.

Our study is not without limitations. First, we included both confirmed and suspected cholera cases in our review. To ensure consistency, WHO encourages countries to use the WHO case definition for reporting of cholera cases. If only laboratory-confirmed cases are reported, the true burden of the disease may not be accurately reflected and therefore limit the implementation of effective cholera control measures if the real extent of the problem is under-recognized [24], [25]. Second, microbiologic confirmation of cases was undertaken in only 12% to 29% of cases in PIDSR annually, and it is likely that some of the suspected cases that we included were not due to cholera. Furthermore, only 26% of suspected outbreaks were microbiologically confirmed with the other outbreaks having no specimens sent, results not recorded or the few specimens sent were negative for any significant enteropathogen. However, very few organisms aside from *V. cholerae* can cause outbreaks of acute watery diarrhea resulting in deaths among adults. Laboratory confirmation using stool or rectal swab cultures are routinely performed in the regional testing center and should be confirmed in the national laboratory referral center. However, a large proportion of the outbreaks occurred in areas far removed from these testing centers and unless an investigation is conducted, Cary-Blair transport media are not routinely available and personnel may not be familiar with the procedure of obtaining rectal swabs in community hospitals or clinics. Microbiologic confirmation of only a small proportion of suspected cholera cases is also reported in other national surveillance systems in resource-limited countries [26]–[28]. Since culture confirmation takes time, delays in identification of outbreaks have been reported as seen in the experience in Papua New Guinea [29]. Third, although conventional culture is the gold standard in the diagnosis of cholera, in a study in Bangladesh, ∼40% of suspected cholera cases occurring during acute diarrhea outbreaks and lacking a confirmed etiology were later determined to have *V. cholerae* using direct fluorescent antibody assay, multiplex-PCR and El Tor-specific lytic phage on plaque assay [30].

Fourth, in adopting the WHO definition, the suspected cases that were identified were primarily in the older age groups; hence we may be missing cholera cases in the youngest age group. The WHO definition aims to maintain specificity, since in children under 5 years of age, a number of pathogens, e.g. rotavirus, can produce symptoms similar to those of cholera [6]. In endemic areas like the Philippines, children under 5 years may have cholera [31] and in a study on rotavirus diarrhea in 2005–2006 among children aged 0–5 years, *V. cholerae* O1 Ogawa was identified from one child who died [32]. Fifth, unless the specimens are obtained early in the onset of the disease, false negative tests may result due to the propensity of antibiotic use in the country, some patients may have taken antibiotics. Conversely, if the specimens were not taken and transported in the right conditions, falsely negative tests will result leading in fewer number of cases. Sixth, there may have been instances when double counting of cases may have occurred. The PIDSR dataset is anonymized and the reports from outbreaks did not have individual information. Furthermore, data from ProMED relied on newspaper reports. However, the dates and locations of the outbreaks from ESR and ProMED were crosschecked with the PIDSR datasets to minimize double counting. Lastly, our data is affected by the limitations of the sentinel surveillance and outbreak reporting. The absence of reports in some provinces may be related to weak surveillance, rather than absence of disease. Sentinel hospitals may not consistently report and are dependent on surveillance staff in the hospitals and clinics. Surveillance staff in the hospitals have additional responsibilities that limit their capacity to report patients on a timely basis. Similarly, all outbreak reports may not be investigated because investigations are dependent on timely reporting to the health department. Although we tried to obtain all ESR reports from 2010 and PIDSR data from 2008, we may have missed some reports and cases.

Despite these limitations, our study provides the most comprehensive picture of cholera in the Philippines in recent years. It is comforting to note that the incidence in our analysis is somewhat similar to the estimates by Ali, et al of 0.1 cases per 1,000 individuals for the Philippines [2]. The figures obtained by Ali et al, however were estimated based on reports that were available from PubMed, ProMED and Gideon databases of cases occurring from 2000 to 2008 that identified only 1,125 cases and 12 deaths in the Philippines and used different methodologies. In our attempt to define the burden of cholera in the country, we identified 42,071 suspected and confirmed cases, substantially more than Ali, et al. The similarity of the estimated incidence published in 2012 to our calculated annual incidence of 9.1 per 100,000 may be due to the inclusion of both suspected and confirmed cholera cases. Our study is the first to ascertain the scope and the burden of cholera in the country. In the future, as the national disease surveillance system matures, more accurate cholera burden estimates may be obtained. More in-depth testing using other microbiologic techniques identifying other etiologies of diarrhea may be conducted and use of other methodologies such as capture-recapture analysis may also be performed.

When cases were mapped at the municipality or city level, the incidences of suspected and confirmed cholera cases were higher in some years than the incidences seen in prospective epidemiologic studies in known cholera endemic areas in other countries [31]. Since cholera tends to affect specific areas, mapping at the municipality or city level will allow public health specialists and local governments to identify areas at risk and thus prepare preventive measures.

Circumscribed outbreaks affecting cities and municipalities appear to occur when there were breaks in the water system particularly in areas where open defecation was practiced. Based on our model, access to improved sanitation was associated with less cholera cases. Paradoxically, access to improved water sources was associated with higher cholera incidence using both suspected and confirmed cholera data sources. This finding may have been likely due to the breakdown in the infrastructure and non-chlorination of water supplies, emphasizing the need for maintenance of public water systems. This finding was supported by the FETP investigations. The majority of the outbreaks that were investigated occurred in areas that had “improved drinking water” sources as defined by the WHO/UNICEF Joint Monitoring Programme (JMP) for Water Supply and Sanitation. According to the JMP, an improved drinking water source is one that “by the nature of its construction and when properly used, adequately protects the source from outside contamination, particularly fecal matter” [33]. Despite construction of water facilities, cholera outbreaks occurred when there were breakdowns in the water systems particularly when these were not appropriately maintained. The breakdown in water facilities may have led residents to use other sources of water such as rivers and streams that were contaminated. This finding was also seen in Sarawak, Malaysia wherein cholera was associated with interruptions in water supply [34].

Similar to other countries in Asia, fear of trade and travel sanctions as a result of cholera being reported in the country remains to be a deterrent in accurate reporting and recognition of the disease. With the change in IHR regulations that specifically calls for global communications and cooperation to allow early detection and mitigation of potential public health events of international concern, it is hoped that more countries will report the occurrence of the disease. This will allow better coordination of disease control measures. Furthermore, with the World Health Assembly resolution in 2011 calling for a coordinated approach to cholera control including the use of OCVs together with water and sanitation [35] the WHO organized an OCV stockpile that can be accessed by countries facing a cholera outbreak [36].

Our findings confirm that cholera is endemic in the Philippines, affecting a large proportion of the provinces in the country. Based on our findings, the DOH is reviewing its guidelines on cholera response in the country including strengthening surveillance and improving response to cholera. Enhanced disease surveillance activities with laboratory support will be necessary to better define the disease burden and will support the development and implementation of policies to minimize the morbidity and mortality due to this dreaded disease.

## Supporting Information

S1 Table Characteristics of the Philippine Integrated Disease Surveillance and Response and the Event-based Surveillance and Response.
(DOCX)

S2 Table Summary of outbreak investigations assessing factors that may have contributed to the outbreaks and recommendations for control.
(DOCX)
