# Supplementary material for: Epidemiology of Cholera in the Philippines
Source: PLoS Negl Trop Dis. 2015 Jan 8;9(1):e3440. doi: 10.1371/journal.pntd.0003440 (PMC4287565; doi:10.1371/journal.pntd.0003440)
Supplement: S1 Table — Characteristics of the Philippine Integrated Disease Surveillance and Response and the Event-based Surveillance and Response. (DOCX) [file pntd.0003440.s001.docx]

Supporting Information

Table S1. Characteristics of the Philippine Integrated Disease Surveillance and Response and the Event-based Surveillance and Response

|  | **Philippine Integrated Disease Surveillance and Response (PIDSR)** | **Event-based Surveillance and Response (ESR)** |
| --- | --- | --- |
| Definition | - Indicator-based surveillance, i.e. routine reporting of cases of disease, including notifiable disease surveillance systems, sentinel surveillance and laboratory based surveillance [1]   Monitors disease morbidity and mortality in order to more effectively guide control programs and the corresponding allocation of resources [2]. | - Rapid detection, reporting, confirmation, assessment of public health events, including clusters of diseases and unexpected deaths [1]   Provides timely information of potential public health threats requiring quick investigation and response and ensures that outbreaks are rapidly detected and controlled [2]. |
| Coverage | - Covers 23 diseases or conditions for surveillance - Consolidated all previous separate surveillance systems including the  1. National Epidemic Sentinel Surveillance System, a hospital based surveillance system that previously monitored 15 diseases with outbreak potential 2. National Disease Reporting System, a field-based surveillance monitoring 17 diseases and 7 syndromes from community, municipality or city health units 3. Expanded Programme on Immunization (EPI) surveillance, an intensive, case-based and hospital-based surveillance of diseases that are being targeted for elimination or eradication [3] | Collection and analysis of ***any*** health event obtained from different media (television, radio, print and internet), health facilities at the different levels of the health system (DOH and other attached agencies, partner agencies and local government units) and from the general public (concerned citizens).  Health events may be actively (through searches on the internet and reviews of different media) or passively obtained (reports by media personnel or local government units) [4] |
| Reporting units | Includes approximately 500 hospitals and clinics | Open reporting, i.e., even the public may report events [4] |
| Case definitions | Definitions used are provided in the PIDSR Manual of Procedures and followed by trained surveillance health care workers [3] | Definitions may be broad, such as a cluster of deaths in the same village during the same time period, to allow increased sensitivity [1]. |
| Timeline | Data are reported every week although some diseases and syndromes may be immediately notifiable e.g. acute flaccid paralysis.  Aggregated data reporting may result in delays in case-reporting. A further delay in reporting may occur with laboratory testing. | All events reported to the Department of Health, ESR unit, are rapidly assessed for the risk the event poses to public health and for appropriate and immediate response. |
| Data/ information collected | Pre-defined information obtained using specific forms used for each disease | Data format is not pre-defined, although complete information is obtained and recorded as much as possible. Staff are trained to obtain key information (e.g. time, place, person) that will assist in confirming and assessing the event. |
| Reporting structure | Reporting begins from sentinel hospitals or clinics to city or provincial health units then to regional health offices and ultimately to the National Epidemiology Center.  Consistency in reporting across the regions varies.  Reporting forms are used by reporting units to submit information through the system on pre-defined days of the week | Reports are unstructured and can enter the system at any time.  Since reports may come from multiple sources, consistency in reporting of health events varies  Forms are used to capture the event information, collects qualitative and quantitative data. |

References:

- 1. WHO (2008) A guide to establishing event-based surveillance. Manila: WHO Regional Office of the Western Pacific.
- 2. WHO. WHO/HSE/GCR /2013 Technical consultation on event-based surveillance, 19-21 March 2013; 2013; Lyon. WHO.
- 3. DOH (2008) National Epidemiology Center. Manual of Procedures for the Philippine Integrated Disease Surveillance and Response. Manila: DOH.
- 4. NEC (2012) Event-based Surveillance and Response (ESR) Annual Report. Manila: DOH.
