# Supplementary material for: Epidemiology of Cholera in the Philippines
Source: PLoS Negl Trop Dis. 2015 Jan 8;9(1):e3440. doi: 10.1371/journal.pntd.0003440 (PMC4287565; doi:10.1371/journal.pntd.0003440)
Supplement: S2 Table — Summary of outbreak investigations assessing factors that may have contributed to the outbreaks and recommendations for control. (DOCX) [file pntd.0003440.s002.docx]

Supplementary Table. Summary of outbreak investigations assessing factors that may have contributed to outbreak

| Location | No. of days of outbreak | No. of cases  n | No. of deaths  n (CFR) | Age range | Water, sanitation and other factors | Control measures and recommendations |
| --- | --- | --- | --- | --- | --- | --- |
| Milbuk, Palimbang Sultan Kudarat (22) | 18 | 132 | 16 (12%) | 2 mos. - 65 yrs (median: 22 yrs.) | Water source: Open dug well that was contaminated | Water chlorination and/or boiling; Construction of pit privy; Personal hygiene |
| Sta. Cruz, Davao del Sur (23) | 57 | 230 | 3 (1.4%) | 1 mo. - 85 yrs. (median: 4 yrs.) | Water sources: Undeveloped springs, mangrove and shallow wells; Water pipes submerged in sewage contaminating water sources | Personal hygiene and food sanitation; Inspection of water pipes |
| Talalora, Western Samar (24) | 19 | 19 | 0 | 3 mos. - 59 yrs. (median: 10 yrs.) | Water source: Springs that flow through pipes, untreated water sources; Eating raw or half-cooked food; No toilets, open defecation | Boiling and/or chlorination of water, Personal hygiene; Improve sewerage system; Provision of toilet facilities |
| Bataraza, Palawan (25) | 49 | 562 | 20 (3.6%) | 1 mo. - 90 yrs. (median: 8 years) | Untreated water sources; river water drank directly by tribes; open defecation practiced by some | Provision of oral and intravenous rehydration supplies; Boiling or chlorination of water, Construction of communal toilets |
| Nabua, Camarines Sur (26) | 69 | 309 | 2 (0.65%) | 3 mos. - 92 yrs. (median: 1yr.) | Untreated water coming from spring box and deep wells located near septic tank, Leaking water pipes; Heavy rainfall 2 weeks before outbreak; Communal faucet (40%), Open defecation (62%) | Provision of oral and intravenous rehydration supplies; Boiling or chlorination of water, Construction of communal toilets |
| Virac, Catanduanes (11) | 69 | 323 | 5 (1.55%) | 1 mo. - 85 yrs. (median: 5 yrs.) | Untreated water from springs, shallow wells and dug holes near river banks; Open defecation in one village, another village with access to improved sanitation at 29% | Boiling and/or chlorination of water, Personal hygiene; Inspection of water pipes |
| Tucamaror, Bongo Island, Parang Maguindanao (27) | 17 | 66 | 2 (3%) | 4 mos. - 45 yrs. (median: 3 y/o) | Water source: Open dug wells and a tank that catches rainwater; Eating fish | Provision of oral and intravenous rehydration supplies; Water chlorination, Personal hygiene; |
| Brgy. Basag T'boli, South Cotabato (28) | 26 | 103 | 2 (2%) | 3 mos. - 84 yrs. (median: 9 yrs.) | Water source: Spring box connected to communal faucet that was not maintained and not chlorinated; river; Heavy rainfall 2 weeks before outbreak started; Not washing hands before eating; Absence of toilet facilities | Provision of oral and intravenous rehydration supplies; Water chlorination, Personal hygiene; |
